# HMCan: a method for detecting chromatin modifications in cancer samples using ChIP-seq data

**DOI:** 10.1093/bioinformatics/btt524

**Published:** 2013-09-09

**Authors:** Haitham Ashoor, Aurélie Hérault, Aurélie Kamoun, François Radvanyi, Vladimir B. Bajic, Emmanuel Barillot, Valentina Boeva

**Affiliations:** ^1^Computer, Electrical and Mathematical Sciences and Engineering Division, Computational Bioscience Research Center (CBRC), King Abdullah University of Science and Technology (KAUST), Thuwal 23955-6900, Saudi Arabia, ^2^Institut Curie, 75248 Paris Cedex 05, France, ^3^INSERM, U900, Bioinformatics and Computational Systems Biology of Cancer, ^4^Mines ParisTech, Fontainebleau 77300, France and ^5^UMR 144 CNRS, Subcellular Structure and Cellular Dynamics

## Abstract

**Motivation:** Cancer cells are often characterized by epigenetic changes, which include aberrant histone modifications. In particular, local or regional epigenetic silencing is a common mechanism in cancer for silencing expression of tumor suppressor genes. Though several tools have been created to enable detection of histone marks in ChIP-seq data from normal samples, it is unclear whether these tools can be efficiently applied to ChIP-seq data generated from cancer samples. Indeed, cancer genomes are often characterized by frequent copy number alterations: gains and losses of large regions of chromosomal material. Copy number alterations may create a substantial statistical bias in the evaluation of histone mark signal enrichment and result in underdetection of the signal in the regions of loss and overdetection of the signal in the regions of gain.

**Results:** We present HMCan (Histone modifications in cancer), a tool specially designed to analyze histone modification ChIP-seq data produced from cancer genomes. HMCan corrects for the GC-content and copy number bias and then applies Hidden Markov Models to detect the signal from the corrected data. On simulated data, HMCan outperformed several commonly used tools developed to analyze histone modification data produced from genomes without copy number alterations. HMCan also showed superior results on a ChIP-seq dataset generated for the repressive histone mark H3K27me3 in a bladder cancer cell line. HMCan predictions matched well with experimental data (qPCR validated regions) and included, for example, the previously detected H3K27me3 mark in the promoter of the DLEC1 gene, missed by other tools we tested.

**Availability:** Source code and binaries can be downloaded at http://www.cbrc.kaust.edu.sa/hmcan/, implemented in C++.

**Contact:** haitham.ashoor@kaust.edu.sa

**Supplementary information:** Supplementary data are available at *Bioinformatics* online.

## 1 INTRODUCTION

ChIP-Seq is a combination of chromatin immunoprecipitation and next-generation sequencing of extracted DNA fragments (Robertson *et al.*, 2007). The ChIP-Seq technique is now widely used for identification of epigenetic marks such as histone variants and different covalent modifications of histone tails (Furey, 2012). Common histone modifications include lysine acetylation, methylation, ubiquitylation and sumoylation, serine and threonine phosphorylation and arginine methylation (Kouzarides, 2007). Histone marks help partitioning the genome into euchromatin, which is accessible for transcription, and heterochromatin. For instance, trimethylation of lysine 9 of histone 3 (H3K9me3) and trimethylation of lysine 27 of histone 3 (H3K27me3) are marks associated with pericentromeric heterochromatin and regions of polycomb-mediated repression (Kharchenko *et al.*, 2011). Also, histone modifications and histone variants are often associated with distinct biological functions. For instance, trimethylation of lysine 36 of histone 3 (H3K36me3) is a mark of transcription elongation; trimethylation of lysine 4 of histone 3 (H3K4me3) marks active or poised promoters; monomethylation of lysine 4 of histone 3 (H3K4me1) together with acetylation of lysine 27 of histone 3 correlates with active enhancers (Kouzarides, 2007). Some marks are narrow and cover 1–10 consecutive nucleosomes (e.g. H3K4me1 or H3K4me3), whereas others (e.g. H3K27me3 and H3K36me3) can cover large genomic regions, from tens to hundreds of kilobases in length.

Although genetic modifications remain the main cause of cancer development, epigenetic modifications may also play a role in cancer development and progression (Esteller, 2007). DNA methylation and/or histone methylation and deacetylation can be observed either as local modifications or along large genomic regions. When regional, these modifications may cause chromatin remodeling and silence expression of most genes in these regions. This phenomenon is often called regional epigenetic silencing (RES) or long range epigenetic silencing (LRES). RES/LRES has been shown to affect gene expression in many cancer types including bladder cancer (Stransky *et al.*, 2006), colorectal cancer (Dallosso *et al.*, 2012; Frigola *et al.*, 2006), breast cancer (Novak *et al.*, 2008) and prostate cancer (Coolen *et al.*, 2010).

Because of the reversible nature of epigenetic modifications, a substantial effort is being made to develop anticancer drugs able to interfere with the activity of enzymes involved in histone modification (Biancotto *et al.*, 2010).

Many tools have been developed to facilitate the analysis of histone modification data obtained with the ChIP-Seq technique. Some tools are designed to detect narrow peaks of type of H3K4me3 (Kharchenko *et al.*, 2008; Rozowsky *et al.*, 2009; Zhang *et al.*, 2008). Other methods are able to identify epigenetic marks covering large genomic regions; this is mostly done through clustering (Zang *et al.*, 2009), gene-by-gene quantification (Hebenstreit *et al.*, 2011), Hidden Markov Models (HMMs) (Qin *et al.*, 2010; Xu *et al.*, 2008) and linear signal–noise models (Xu *et al.*, 2010).

However, there is no tool specifically developed to detect histone modifications in cancer genomes that takes into account copy number alterations. As we show later, most of the tools tend to detect more signals in the regions of gain and less signal in the regions of loss.

GC-content is known to influence read depth in both Illumina- and SOLiD-generated datasets (Boeva *et al.*, 2011; Dohm *et al.*, 2008). A possible difference in GC-content dependencies between ChIP and control datasets can result in false predictions of enrichment in histone modification marks (Chen *et al.*, 2012).

Here we present a tool designed to identify histone modifications in genomes with large copy number alterations. HMCan (Histone Modifications in Cancer) corrects for copy number bias and for GC-content bias. It then uses HMMs to detect regions rich in histone modifications.

We chose to compare HMCan with three tools commonly used to detect histone modifications with CHIP-seq data: CCAT (Xu *et al.*, 2010), MACS (Zhang *et al.*, 2008) and SICER (Zang *et al.*, 2009). We show that HMCan is able to detect signal enrichment in simulated cancer genomes better than these three tools. Only HMCan and CCAT did not show copy number bias. Separately, on an experimental ChIP-seq dataset of H3K27me3 in a bladder cancer cell line, HMCan provided better results than CCAT.

## 2 METHODS

### 2.1 HMCan algorithm

**Fig. 1.**
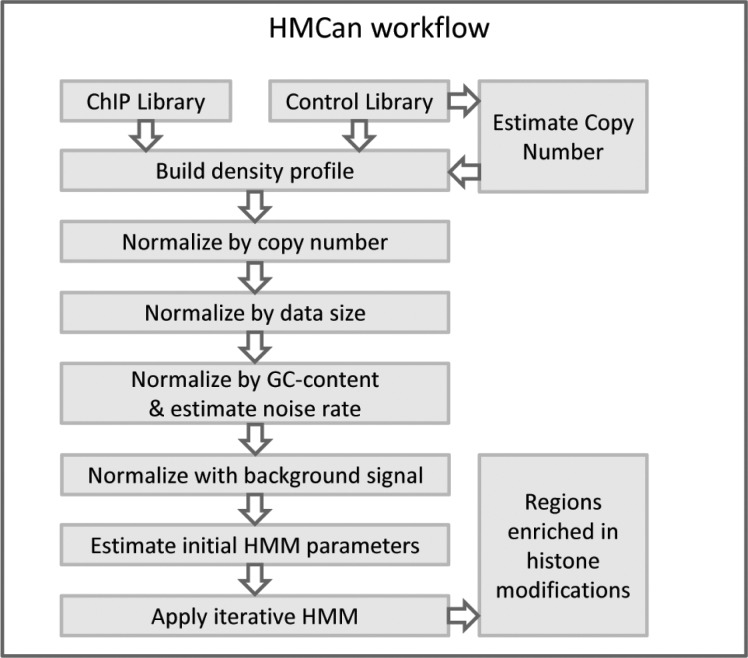
HMCan framework. HMCan initially estimates copy numbers from control data and it builds density profiles for both libraries. Then, HMCan performs a set of normalization steps including normalization by copy number, GC-content and background noise. Finally, HMCan detects histone modification regions with HMMs after estimating initial parameters from the normalized profile

The HMCan workflow consists of (i) estimation of the copy number profile using a window approach on the control dataset (usually, input DNA), (ii) calculation of the density profile, (iii) normalization of the density profile by copy number, GC-content and background signal and (iv) application of HMMs to detect regions with histone modifications (Fig. 1).

#### 2.1.1 Data profile construction

Reads of ChIP and control datasets are transformed into density profiles. To construct the profiles, the reads are extended from read starts to the length of DNA fragments. Similarly to FindPeaks, we use the triangular distribution for read extension (Fejes *et al.*, 2008). This method allows the user to set minimum median and maximum fragment length used in the original ChIP-seq experiment. After read extension, we keep one density value for each 50 nucleotides (this value can be changed by the user).

#### 2.1.2 Correction for copy number

To estimate the copy number variations in ChIP-seq data, we apply the algorithm implemented in Control-FREEC (Boeva *et al.*, 2011, 2012a). When the copy number of each position is estimated, each value in the density profile is corrected based on its copy number value.

#### 2.1.3 Data size correction

Assuming that the ChIP dataset contains *N* reads and the control dataset contains *M* reads, the ChIP density profile is multiplied by the ratio between these numbers (*M*/*N*).

#### 2.1.4 Initial peak calling

To calculate the correct GC-content profile on the ChIP data and correctly estimate the initial parameters of HMM, preliminary peak (enrichment signal) calling should be applied to serve as a guide for both operations. A one-sided exact Poisson test is used to label whether a bin belongs to a peak or not.

As a post-processing step, singleton bins labeled as peaks are removed. Then, the bins labeled as peaks within 1 Kb are merged into a single peak region.

#### 2.1.5 GC-content normalization

Sequencing technologies may result in association between number of reads mapped to a specific DNA region and its GC-content (Benjamini and Speed, 2012). Here, we apply a correction to remove GC-content bias, which otherwise may result in aberrant read counts.

We estimate the GC-content bias from the density profiles previously constructed. For each value of bin density, we take a window of length twice that of the fragment length. With each window, we associate the density value corresponding to the central point and we record the value of the GC-content of that window.

GC-values are grouped in non-uniform groups, e.g. GC-content between 0 and 20% (group 1), GC-content between 20 and 22% (group 2), and so forth. (Supplementary Methods). For each value *gc* in the group, we will define *D_gc_* – the sum of densities of the bins that have GC-content *gc* and *N_gc_* the total number of windows that have GC-content *gc*. We denote the expected density for each *gc* value as **λ***_gc_*, defined as:
(1)
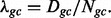

We will denote the average expected density along the genome by **λ,** defined as:
(2)
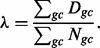

Then, each density value *D* can be corrected as follows:
(3)
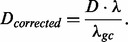

The correction process is applied to both ChIP and control data independently. This leads to a more accurate correction compared with calculating GC-content bias (**λ** and **λ***_gc_*) for the control data only and then correcting the ChIP and control densities based on the same **λ** values.

Applying the described method to the control data is straightforward, as the control data are not supposed to contain any signal. In the case of ChIP data, the process is trickier because the signal contained in the ChIP data may interfere with the GC bias, e.g. some histone modifications can occur more frequently in GC-rich regions. To overcome this issue, we first apply the module of initial peak calling to identify regions that most probably belong to the signal (‘peaks’). Then, we apply the described method for CG-bias evaluation to the regions labeled as ‘not peaks’.

We denote the expected density λ in the control data as λ*_control_* and λ in the ChIP data as λ*_ChIP_*.

To get the noise values in the ChIP and control data on the same scale, we multiply the values of density in the control by the noise ratio λ*_noise_*, where:
(4)
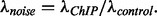

To calculate the final density profile for the ChIP sample, we apply the following normalization:
(5)
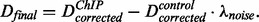



#### 2.1.6 Initial estimation of HMM parameters

HMM is used at the final stage for peak calling. The motivation behind using HMM is that this approach is able to call wide peaks regardless of the noise that may be present. Such large peaks can correspond to RES/LRES, and thus the HMM approach is preferable. Moreover, the HMM approach allows the calling of narrow peaks if their signals are relatively strong. Thus, this approach will not miss short regions with epigenetic changes, e.g. signal present at the Transcription start sites (TSSs) of repressed genes.

The designed HMM has two states: ‘peak’ (1) and ‘not peak’ (0). The description of HMM can be found in Supplementary Methods. The first step in estimating HMM parameters and inputs is to re-call the peaks after all normalization steps using a one-sided Poisson test.

The transition probabilities of HMM are estimated by counting four possible combinations of the states. The emission probabilities are derived from the distributions of the normalized densities over the peak and non-peak data independently.

#### 2.1.7 Iterative HMMs

To infer the correct states along the genome, we use the Viterbi algorithm (Viterbi, 1967). The Viterbi algorithm can decode most of the states from the first run based on the estimated parameters. We noticed that for our data, predictions obtained by the first run contained a substantial amount of noise and predicted regions were not as large as we expected. To overcome these two shortcomings of the Viterbi algorithm, we introduced the iterative Viterbi algorithm, which results in predictions corresponding to longer regions containing less noise.

We iteratively use the following procedure. Each region associated with a peak state has a score S, where S is the Bayesian log-likelihood ratio:
(6)
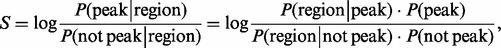

where the probabilities are calculated based on the peak and density distributions observed at the previous step. After calculating *S* for each putative peak, we consider regions with scores less than S_0_, the minimum score to accept the current peak in the next iteration, as ‘non-peaks’. Then, the emission and transition probabilities are recalculated based on the new set of regions. The process of recalculating emission and transition probabilities is identical to the one used for the evaluation of initial parameters. The algorithm keeps iterating until no improvement is noticed or some maximum number of iterations is reached.

Finally, at the post-processing step, peaks within 1 Kb are merged into a single region.

We also provide an option to calculate posterior probabilities for each bin. HMCan calculates posterior probability using forward–backward algorithm given the normalized density value at each bin.

### 2.2 ChIP assay

The human bladder cancer cell line CL1207 was derived from a muscle-invasive bladder cancer (De Boer *et al.*, 1997). CL1207 was cultured in Dulbecco’s modified Eagle medium F-12 GlutaMAX™ (Invitrogen, Cergy Pontoise, France) supplemented with 10% fetal bovine serum (Lonza Verviers, Verviers, Belgium). One confluent 75 cm^2^ dish of CL1207 was used for each ChIP-seq experiment.

CL1207 chromatin was extracted from cell nuclei and sheared enzymatically using an Active Motif kit (Active Motif, Rixensart, Belgium). An extract of the original chromatin was kept as an internal standard (input DNA). The 5 × 10^5^ cells were immunoprecipitated per ChIP assay with 4 μg of rabbit polyclonal antibodies against trimethyl histone H3 lysine 27 (Upstate Biotechnology, Santa Cruz, CA) and Dynabeads® Protein A (Invitrogen, Cergy Pontoise, France) in dilution buffer containing 1% Triton X-100, 150 mM NaCl, 2 mM EDTA, 20 mM Tris–HCl at pH 8.0 and protease inhibitors. Six ChIP assays in the same experimental conditions were necessary to perform one ChIP-Seq experiment, so we used the total of 6 × 10^6^ cells.

### 2.3 ChIP-seq library and SOLiD sequencing

The SOLiD System 2.0 workflow for the lower input/lower complexity DNA fragment library preparation kit was used following the manufacturers’ instructions (Applied Biosystems) starting with 50 and 58 ng of ChIP or input DNA, respectively.

ChIP-seq DNA fragment libraries were sequenced using the SOLiD 5500 system to produce 75-bpreads. The sequencing reads were aligned to the hg19 human genome using Bowtie 0.12.8 (Langmead *et al.*, 2009) with the following options: ‘-C -k 1 -y –col-keepends’.

### 2.4 Gene annotation for ChIP-seq data

To assign predicted H3K27me3 marks to genes, we used the annotation tool included in the Nebula pipeline (Boeva *et al.*, 2012b). A mark was assigned to a gene (RefSeq Release 50; 34 062 gene isoforms), if it overlapped the region 1000 bp upstream and 1000 bp downstream of the gene TSS (Young *et al.*, 2011).

## 3 RESULTS

### 3.1 Evaluation on simulated data

To investigate the performance of HMCan on cancer samples, we constructed a simulated ChIP-seq dataset for a fictional histone mark. The signal covered multiple regions across chromosome 1 (human genome, hg19), with each region being of length from 1 to 20 Kb. These regions comprised 5% of chromosome 1. We simulated histone marks covering different numbers of alleles in the regions of normal copy number, gain (of copy number) and loss (of copy number) (Supplementary Table S1). In our simulations, we set read length = 76 bp, fragment length = 150 bp, ∼20% of the reads came from the signal regions and ∼80% of the reads came from the non-signal regions. We assumed a constant length of DNA around one nucleosome to be equal to 185 bp. We simulated more errors at the end of reads using the standard Illumina error distribution. As sequencing depth depends on GC-content, we used the experimentally observed GC-content dependency function from the ENCODE dataset for the MCF-7 cell line (input and H3K9me3). The code for read generation together with parameters used and necessary files can be found at the HMCan webpage (package ‘GenerateReadsChIP-seq’). Generated reads were aligned to the reference genome with BWA (Li and Durbin, 2009) using default parameters.

To quantify the quality of predictions of HMCan and other tools, we calculated overlap between the predicted regions and the simulated regions at the base pair level. If a base pair within a predicted region overlapped with a simulated one, this base was counted as true positive. If it lay outside of the simulated region, it was counted as false positive. Finally, if a base pair within a simulated region was not covered by any prediction it was counted as false negative. Next, recall the definitions of *recall* and *precision* as:
(6)
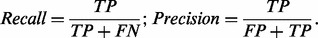



The recall measures the sensitivity of a prediction method, whereas precision measures the proportion of true predictions within all positively predicted regions. In cases where the number of true negatives is large, it is advisable to use ‘precision *vs* recall’ curves instead of standard ROC curves (‘recall’ versus ‘false positive rate’) (Davis and Goadrich, 2006), for more details check (Supplementary Methods). In our case, the number of TN is large because the true signal covers a small fraction of the genome (5%).

**Fig. 2.**
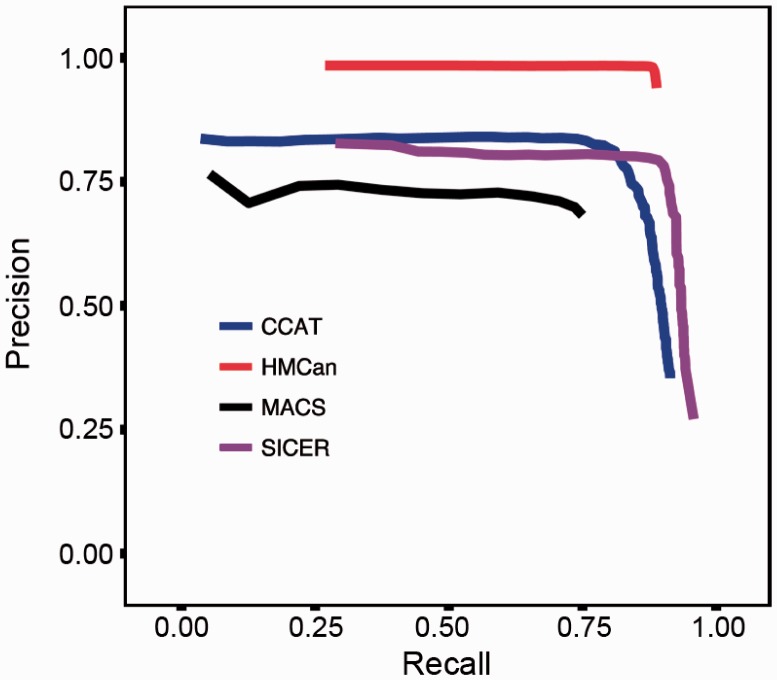
Relationship between *recall* and *precision* for HMCan and other histone modification detection tools on the simulated data. HMCan shows higher prediction accuracy compared with other tools and a noticeable difference in the precision

On the simulated data, HMCan demonstrated a better prediction accuracy than three tools commonly used to detect histone modifications with ChIP-seq data: CCAT (Xu *et al.*, 2010), MACS (Zhang *et al.*, 2008) and SICER (Zang *et al.*, 2009) (Fig. 2). CCAT applies an iterative method to estimate the noise-to-signal ratio in ChIP-seq and control data based on a linear model. MACS shifts the reads toward the fragment centers and uses a dynamic Poisson model that is able capture the mean and standard deviation of the data. SICER applies a read clustering approach to detect regions enriched with histone marks. For each tool, we ranked the predicted regions according to the in-built score or *P*-value and grouped them in sets of regions having similar scores. By using a threshold on this score or *P*-value, we obtained ‘precision *vs* recall’ curves. The accuracy of predictions was qualified on the basis of the closest (Euclidian) distance from the ideal predictor performance as introduced in Bajić (2000), which in our case is the distance from the (1,1)-corner of the ‘precision *vs* recall’ graph (Fig. 2). To make the comparison fair, we checked several combinations of parameters of other tools such as CCAT (Supplementary Fig. S1) and SICER (Supplementary Fig. S2). The best parameters for CCAT were: minScore = 2, window = 1000; for SICER: Gap = 600.The result corresponding to the best combination of parameters is shown in Figure 2. With the best configuration of parameters, HMCan was able to identify 88.4% of base pairs within simulated signal regions, and its positive predictions contained only 3% of false-positive predictions at the base pair level (Table 1). CCAT, MACS and SICER achieved lower accuracy than HMCan. Generally, SICER demonstrated a high sensitivity of predictions (recall = 87.4%) together with a considerable false discovery rate (Precision = 79.8%). CCAT showed high precision (81.1%), being second only to HMCan, but failed to detect a large part of the signal (recall = 81%).

**Table 1.** HMCan provides better accuracy of predictions than CCAT, MACS and SICER on simulated data

Method	Best precision		Best recall		Best combination	
	Precision	Recall	Precision	Recall	Precision	Recall
HMCan	**0.984**	0.493	0.939	0.888	**0.971**	**0.884**
CCAT	0.841	0.578	0.354	0.913	0.811	0.810
MACS	0.766	0.052	0.682	0.750	0.699	0.735
SICER	0.828	0.288	0.271	**0.958**	0.798	0.874

*Note*: The ‘best combination’ corresponds to the shortest distance from the ideal predictor performance. The results of the best performing methods are shown in bold.

We assessed sensitivity of the HMCan’s iterative HMM method to the change of the initial parameters (i.e. threshold on the *P*-value of the exact Poisson test). We reported high values of Jaccard similarity index between predictions corresponding to different *P*-value thresholds (>0.97, see Supplementary Methods and Supplementary Table S2). The corresponding ‘Precision vs Recall’ curves (Supplementary Fig. S3) for different *P*-value thresholds also confirm that the final predictions are not influenced by the initial threshold setting.

**Fig. 3.**
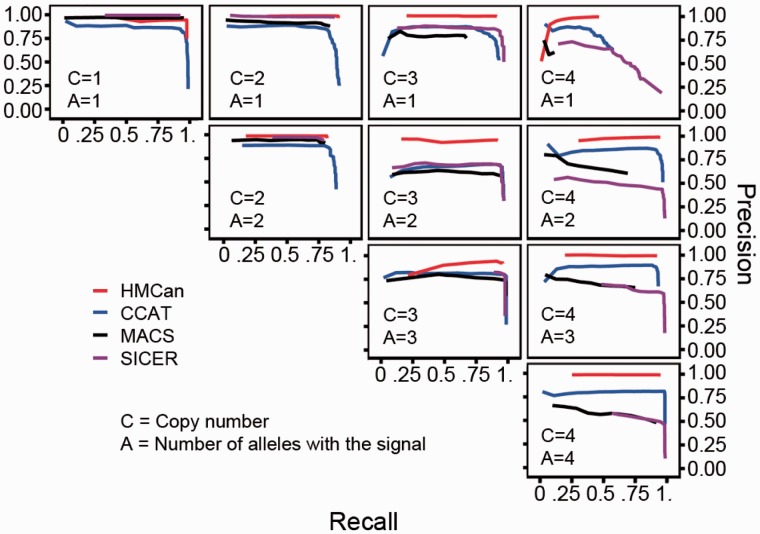
Relationship between *recall* and *precision* for HMCan and other histone modification detection tools on simulated data sub regions associated with different copy number status and signal

We explored what combination of copy number status and number of alleles with histone modification signal was the most challenging for histone modification detection (Fig. 3). As expected, for all tools it was more difficult to detect the correct regions in the situation when only one allele out of four was bearing a histone modification mark (Fig. 3: C = 4, A = 3). In this extreme situation, SICER demonstrated the best sensitivity compared with other tools. However, the best combination of recall and precision was achieved by HMCan. Interestingly, accuracy of predictions of SICER and MACS highly depended on copy number (Fig. 3, diagonal panels with A = C). For instance, the precision values of SICER’s and MACS’ predictions were close to one when the signal was present in one allele out of one or in two alleles out of two (Fig. 3, A = C = 1 and A = C = 2). When the signal was present in three alleles out of three or in four alleles out of four, both MACS and SICER predicted more signal than it was put to the simulated data (Fig. 3, A = C = 3 and A = C = 4).

HMCan and CCAT did not demonstrate such copy number bias. Thus, we conclude that these two tools are the most suitable for histone modification signal detection in cancer data.

### 3.2 Evaluation on H3K27me3 data

**Fig. 4.**
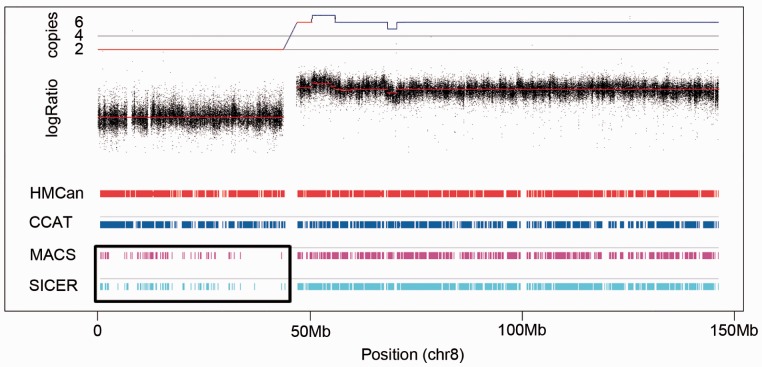
Predictions of SICER and MACS are biased toward regions of genomic gain, whereas predictions of CCAT and HMCan do no show copy number bias. Top track: copy number profile for chromosome 8 of the CL1207 human bladder transitional cell carcinoma cell line calculated by GAP (Popova *et al.*, 2009) using SNP array technology; Bottom tracks: regions predicted to have the H3K27me3 mark by HMCan, CCAT, MACS and SICER. The black frame shows the chromosome arm 8p, which has lower density of sites predicted by MACS and SICER

To assess the performance of HMCan on real data, we generated ChIP-seq dataset for trimethylation of lysine 27 on histone H3 (H3K27me3) for the CL1207 human bladder transitional cell carcinoma cell line (see Materials and Methods). We compared HMCan and CCAT on this dataset. As the MACS and SICER tools demonstrated a bias toward high copy number regions (Figs 3 and 4), the comparison of HMCan with MACS and SICER is given in Supplementary Materials (Supplementary Figs S4 and S5 and Supplementary Table S3).

**Fig. 5.**
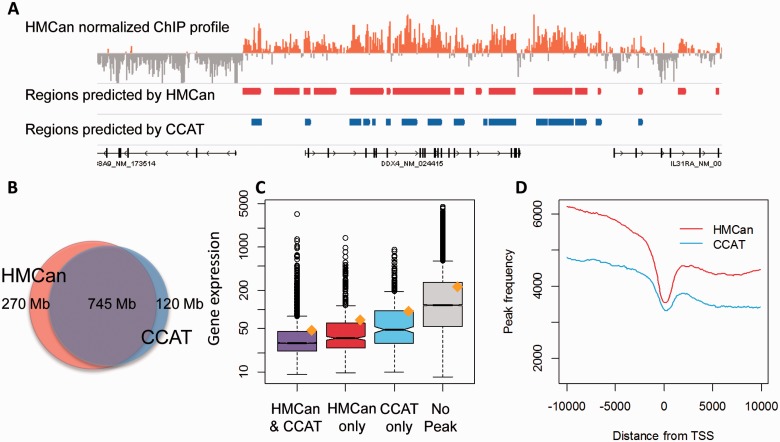
Comparison of HMCan and CCAT on the H3K27me3 ChIP-seq dataset for the CL1207 (bladder cancer) cell line. (**A**) Predicted regions as well as normalized density are visualized with Integrated Genome Browser (IGV) (Robinson *et al.*, 2011; Thorvaldsdóttir *et al.*, 2012); (**B**) Venn diagram showing base pair overlap in the HMCan and CCAT predictions, numbers show the total number of nucleotides in predicted regions, Jaccard similarity coefficient 0.66; (**C**) Genes with an H3K27me3 mark predicted by HMCan in the promoter regions (TSS ± 1 kb) tend to have lower expression than genes with this mark predicted by CCAT. The graph shows the distribution of gene expression values after RMA normalization for the following gene categories: genes with the promoter H3K27me3 mark predicted by (i) both HMCan and CCAT, 3846 genes, (ii) HMCan only, 635 genes, (iii) by CCAT only, 391 genes and (iv) by none of the tools, 12 826 genes. The orange dot shows the mean expression value. The black line shows the median and the boxes are plotted between the first and the third quartiles; (**D**) Density of peaks detected by HMCan and CCAT around all gene TSSs for the H3k27me3 histone mark in the CL1207 cell line

To detect the H3K27me3 mark, we ran HMCan and CCAT with the parameters learned from the simulated data study. We considered all regions detected by HMCan or CCAT regardless of the score (see justification in Supplementary Methods). Overall, 32.8 and 28% of the genome were covered by regions predicted by HMCan and CCAT, respectively. There was a large overlap in the predictions (Fig. 5A and B). Further, we will show that genomic regions, predicted to bear the repressive H3K27me3 mark by HMCan only, are unlikely to be false-positive predictions. We will demonstrate that such regions, when falling within gene promoters, suggest lower gene expression. Also, the profile of HMCan predictions around gene TSS has a relatively more prominent valley at TSS than the profile of CCAT predictions. Finally, we will show that predictions of HMCan are more accurate for a set of qPCR validated H3K27me3 regions in the CL1207 cell line.

We studied the correlation between gene expression and H3K27me3 predictions by HMCan and CCAT in promoter regions. We used gene expression values calculated from exome arrays (unpublished data) for the CL1207 cell line. Normalization was performed with the robust multiarray averaging (RMA) method to get exon expression signals; then the median over exon signals was calculated to get a signal value per gene. H3K27me3 is a repressive histone mark associated with DNA methylation of CpG islands in gene promoters (Ku *et al.*, 2008). Thus, we expected the genes with the real H3K27me3 mark in promoter (TSS ± 1 Kb) to have lower expression than genes without H3K27me3 in the promoter. Indeed, genes for which none of the tools predicted an H3K27me3 site had higher expression than genes with H3K27me3 predicted by both CCAT and HMCan (Fig. 5C; one-tailed Mann-Whitney test, *P*-value < 10^−^^16^, median values 117.94 versus 29.24). Interestingly, expression values of genes with H3K27me3 predicted by HMCan only were significantly lower than expression values of genes with H3K27me3 predicted by CCAT (Fig. 5C; one-tailed Mann-Whitney test, *P*-value 5.1 × 10^−^^10^, median values 35.02 versus 47.98). This result indirectly shows that HMCan generally predicts stronger H3K27me3 sites that CCAT. However, this result does not reject the hypothesis that CCAT-only predictions may correspond to weaker but true H3K27me3 sites. Only slightly more of the HMCan-only predictions fall within the copy number alteration regions as compared with the CCAT-only predictions: 60.3% versus 57.6%, respectively (see Supplementary Materials and Supplementary Fig. S6 for more detail).

We calculated the H3K27me3 signal distribution around TSSs of coding genes (RefSeq Release 50; 34 062 gene isoforms). For both HMCan and CCAT, we added to the density counts nucleotide positions covered by the predicted regions. Generally, the H3K27me3 mark exhibits a decreasing profile from 5′ to 3′ with a pronounced valley in the vicinity of TSS (Barski *et al.*, 2007; He *et al.*, 2012). We observed the expected profile in regions predicted by HMCan and, to a slightly lower extent, in the predictions of CCAT (Fig. 5D).

In our previous study (Vallot *et al.*, 2011), we validated by qPCR several gene regions bearing the repressive H3K27me3 mark in the CL1207 cell line. HMCan successfully detected H3K27me3 marks on the DLEC1 gene, which is commonly deleted in various carcinomas (Chan *et al.*, 2010; Ying *et al.*, 2009), as well as on the homeobox D (*HOXD*) gene cluster located at 2q31-2q37 chromosome regions. The *HOXD* cluster includes genes *HOXD1*, *HOXD3*, *HOXD4* and *HOXD8-13*. Many of these genes have been shown to play a crucial role in oncogenesis (Shah and Sukumar, 2010). Low expression of several *HOXD* genes was detected in neuroblastoma (Manohar *et al.*, 1996; Zha *et al.*, 2012), breast (Carrio *et al.*, 2005) and colorectal (Jung *et al.*, 2005) cancer. Interestingly, although CCAT successfully detected H3K27me3 on the *HOXD* cluster, it failed to identify the H3K27me3 mark in the promoter of *DLEC1* [HMCan peak score 0.25, relative enrichment assessed by ChIP-qPCR 0.22 (Vallot *et al.*, 2011), Supplementary Fig. S7 and Supplementary Table S3].

## 4 DISCUSSION

We have developed HMCan, a tool for detection of histone modifications in cancer samples using ChIP-seq data. On simulated data, HMCan demonstrated better accuracy of prediction compared with the other tools we tested.

HMCan was originally developed to identify broad signals such as H3K27me3 or H3K36me3. However, it can be also applied to a class of histone modifications with narrow signal (e.g. H3K4me3). The output of HMCan includes information about peak maxima to facilitate functional annotation of peaks. The output also includes normalized density profiles (Fig. 5A, top), which is convenient for inspecting predicted regions with histone marks and can be also used for producing figures for publication.

The run time of HMCan is significantly longer than for the majority of other tools (e.g. MACS, CCAT or SICER) and may require up to 1 h on a standard PC (Supplementary Table S4). Running iterative HMMs is the most time-consuming step. However, we believe that the greater accuracy of HMCan demonstrated in this study compensates for a longer run time.

In some cases, it might be interesting to detect differential histone modifications using two ChIP-seq datasets generated for two different conditions. HMCan does not as yet contain such a function, and we advise users to apply standard tools such as DESeq (Anders and Huber, 2010) to the output from HMCan.

## 5 CONCLUSION

HMCan was specifically developed to analyze histone modification data obtained for cancer genomes. Cancer genomes are characterized by frequent copy number alterations: gains and losses of large regions of chromosomal material. The designed algorithm explicitly corrects for copy number alterations, and thus does not demonstrate bias within the number of predicted sites in the regions of gain or loss. In addition, HMCan corrects for possible GC-content bias independently in the ChIP and control sample. This guarantees GC-content-unbiased results even in the case when the two experiments are performed in different laboratories and even using different sequencing techniques. Also, the iterative HMM method formulated in HMCan allows for getting the best distinction between signal and noise on sequential data. HMCan accepts the most common alignment formats: SAM/BAM and BED, and outputs predicted regions in BED and WIG formats.

We successfully applied HMCan on both simulated and experimental data. On simulated data, we demonstrated HMCan’s higher accuracy in signal prediction compared with the tools designed for normal genomes (MACS, SICER and CCAT). Unlike MACS and SICER, HMCan did not show bias in number of identified peaks toward gained regions. In our simulations, we modeled signal in regions present in 1, 2, 3 or 4 copies. HMCan detected the signal in all of them, including cases where the signal was initially present in only one out of four alleles. On the experimental ChIP-seq dataset generated for the repressive mark H3K27me3 in the CL1207 human bladder transitional cell carcinoma cell line, peaks in proximity of gene TSSs that were detected only by HMCan corresponded to lower gene expression compared with the peaks detected only by CCAT. Overall, HMCan proves to be an appropriate tool for predicting histone modifications in genomes with copy number alterations.

## Supplementary Material

Supplementary Data
