# Supplementary material for: HMCan: a method for detecting chromatin modifications in cancer samples using ChIP-seq data
Source: Bioinformatics. 2013 Sep 9;29(23):2979–86. doi: 10.1093/bioinformatics/btt524 (PMC3834794; doi:10.1093/bioinformatics/btt524)
Supplement: Supplementary Data [file supp_29_23_2979__index.html]

HMCan: a method for detecting chromatin modifications in cancer samples using ChIP-seq data — HMCan: a method for detecting chromatin modifications in cancer samples using ChIP-seq data — HMCan: a method for detecting chromatin modifications in cancer samples using ChIP-seq data — Supplementary Data 

# HMCan: a method for detecting chromatin modifications in cancer samples using ChIP-seq data

## Supplementary Data

files

**Files in this Data Supplement:**

- Supplementary Data - pdf file
