# Supplementary material for: HMCan: a method for detecting chromatin modifications in cancer samples using ChIP-seq data
Source: Bioinformatics. 2013 Sep 9;29(23):2979–86. doi: 10.1093/bioinformatics/btt524 (PMC3834794; doi:10.1093/bioinformatics/btt524)
Supplement: Supplementary Data [file supp_btt524_Ashoor_Supplementary_Materials.pdf]

Supplementary Materials

HMCan: a method for detecting chromatin modifications in cancer samples using ChIP-seq data

Haitham Ashoor, Aurélie Hérault, Aurélie Kamoun, François Radvanyi, Vladimir B. Bajic, Emmanuel Barillot and Valentina Boeva

Table of Contents

Supplementary Methods **1**
- **GC-content normalization** 1
- **HMM description** 1
- **Evaluation of the prediction accuracy on simulated data** 2
- **Stability of the iterative HMM method** 2
- **Evaluation of the prediction accuracy on H3K27me3 ChIP-seq data for the CL1207 human bladder transitional cell carcinoma cell line** 3

Supplementary Figures **5**
Supplementary Tables **13**

Supplementary Methods

GC-content normalization

In order to normalize the data for the GC-content bias, we need to construct dependency function between GC-content values and the expected read density for each GC-content value. We define GC-content as the percentage of G and C nucleotides per window.

Depending on the number of windows that exhibit certain GC-content (Suppl. Figure 8), the dependency function may have some deviations from the bell shape, especially in the regions of high and low GC content (Suppl. Figure 9). We use a non-uniform binning to overcome this problem. This results in a smooth bell shaped distribution of the dependency function (Suppl. Figure 10).

HMM description

A Hidden Markov Model (HMM) is defined by ***H* = {*S*, *O*, *T*, *E*}**, where ***S*** is the set of possible states, **S = {Peak, Not peak}**, ***O*** is the set of observations, ***T*** is the set of transition probabilities, and ***E*** is the set of emission probabilities. Below, we describe ***H* = {*S*, *O*, *T*, *E*}** created by HMCan.

S is the set of possible states. Our model can identify 2 states:

- **Peak**, in which the bin corresponds to a region enriched in histone modifications,
- **Not peak**, in which the bin corresponds to the background.

Of note, we do not define more potential states, e.g., states for monoallelic histone modifications. The main reason is that the monoallelic histone modification can be effectively captured with the two state model (Figure 3B, 3D, 3H). And even though from the biologist's point of view it can be useful to detect monoallelic events, in practice we are not able to distinguish between a strong monoallelic signal and a weak signal involving both alleles.

O is the set of the observations. We define ***O*** as a set of values that can be taken by the normalized ChIP densities at each bin (D_{final} in the main text, formula (5)). $\boldsymbol{O} \subseteq \mathbb{R}$. In general, higher values of densities correspond to the "Peak" state, while lower values of densities correspond to the "Not peak" state.

Transition probabilities ***T*** and emission probabilities ***E*** are derived from the empirical distribution of the data. To initialize ***T*** and ***E***, we fist classify each bin as belonging to one of the two states - "Peak" or "Not peak" - using a one-sided exact Poisson test. For ***T*,** we count frequencies of four possible combinations of transitions between our states. ***E*** is derived from the observations as a conditional empirical distribution of the observations given their initial annotation of state. ***T*** and ***E*** are recalculated at each round of the iterative procedure based on the state annotation at the previous step.

Evaluation of the prediction accuracy on simulated data

In order to investigate the performance of HMCan on cancer samples, we constructed a simulated ChIP-seq dataset for a fictional histone mark. We tested HMCan, CCAT, SICER and MACS using the simulated dataset. The criterion of performance was high precision and high recall at the base-pair level. We tried different options for CCAT and SICER, since these two tools were sensitive to the choice of parameters (Suppl. Figure 1 and 2). The best parameters for CCAT were: minScore = 2, window = 1000; for SICER: Gap = 600.

To evaluate accuracy of predictions, Davis and Goadrich (2006) advice using "Precision vs Recall" curves instead of standard ROC curves ("Recall" *vs* "False Positive Rate") when the number of true negatives is large. We used the distance from (Precision=1, Recall=1) as a measure of the accuracy for the tools.

Stability of the iterative HMM method

The iterative HMM method requires a good guess of the initial parameters to perform well. In HMCan, the initial guess is based on the one-sided exact Poisson test for each bin: If the Poisson test p-value is lower than a threshold, we label this bin as "Peak"; otherwise, we assign to the bin a label "Not peak". The emission and transition probabilities are further calculated using this labeling.

We checked the stability of the iterative HMMs method starting from different thresholds on the Poisson test p-value. We explored four different starting points corresponding to thresholds of 0.01, 0.03, 0.05, and 0.08.

We use Jaccard similarity index as measure of the iterative HMMs stability. Jaccard index in this case can be represented as:

$$J(A,B) = \frac{A \cap B}{A \cup B},$$

where A and B are two set of peaks coordinates resulting from two different thresholds. The operations are done on base pair level. Jaccard index is an indicator how similar the two groups of coordinates are.

Pairwise comparison of HMCan results obtained with different initial parameters demonstrated the limited influence of the initial parameters on final predictions. The Jaccard similarity index between the predictions obtained with different starting points was greater than 0.97 for any pair of p-value thresholds (Suppl. Table 2).

Also, we calculated "Precision vs Recall" curves for the four p-values thresholds to see whether prediction accuracy changes depending on the initial parameters of HMM (Suppl. Figure 3). We conclude that the accuracy of the predictions does not depend on the initial settings of the Poisson p-value threshold.

Evaluation of the prediction accuracy on H3K27me3 ChIP-seq data for the CL1207 human bladder transitional cell carcinoma cell line

Comparison of HMCan and CCAT.

We run CCAT and HMCan with parameters learn from the simulation study, i.e. HMCan: smallBinLength 50, largeBinLength 100000, pvalueThreshold 0.01, mergeDistance 1000, iterationThreshold 2, finalThreshold 0, maxIter 10; CCAT: minScore 2, window 1000, movingStep 50, minCount 2, bootstrapPass 50.

We notice a high overlap in predictions of HMCan and CCAT (Figure 4A, 4B). On simulated data, the "best" thresholds for the internal peak score of HMCan and CCAT were 10 and 3.35, respectively. After applying these thresholds to the predictions of both tools on the H3K27me3 dataset, we obtained Jaccard similarity coefficient equal to 0.38 (Suppl. Figure 11). Note, if we don't apply any thresholds to the predictions of HMCan and CCAT, the observed overlap is larger: Jaccard index 0.66. Often, a region predicted by one tool (e.g., CCAT) with a score less than the "best" threshold (< 3.35) is predicted by the other tool (HMCan) with a score higher than the "best" threshold (> 10) (Suppl. Figure 12).

Thus, we decided not to use thresholds for further comparison of CCAT and HMCan results. Actually, looking at the (Suppl. Figure 12); it is impossible to select a threshold discriminating outlier predictions of CCAT and HMCan. We keep all predictions regardless the threshold as positives for further analyses.

Also, we checked how well HMCan corrects for copy number as applied to the H3K27me3 ChIP-seq data. Thus, we compared the two log-ratio profiles: one generated by the GAP software (Popova et al., 2009) (Suppl. Figure 13) and one evaluated by HMCan using the input reads of the ChIP-seq experiment (Suppl. Figure 14). The Pearson correlation coefficient between log-ratio values for segments independently predicted and analyzed by GAP and HMCan was 0.8963. We thus concluded that the FREEC module of the HMCan method was capable to successfully identify most of the copy number changes.

Since on the experimental H3K27me3 data HMCan outperformed CCAT, we checked whether it was due to the fact that one of the tools tends to detect more sites in the copy number alteration regions

compared to the other tool. We selected genes that had an H3K27me3 mark in the promoter predicted by HMCan only (HMCan-specific genes) and genes that had an H3K27me3 mark in the promoter predicted only by CCAT (CCAT-specific genes). We defined regions of gain and loss as regions that have a log-ratio greater than 0.1 or smaller than -0.1 in the GAP predictions for the CL1207 cell line. Such regions cover in total 60.4% of the genome. We observed only a slightly higher proportion of HMCan-specific genes fell in the regions of gain and loss (60.3%) compared to the CCAT-specific genes (57.6%). This difference was not statistically significant (Fisher's Exact Test p-value 0.41). Overall, distributions of log-ratio values corresponding to HMCan- and CCAT-specific genes were similar to the distribution of log-ratio values for all RefSeq genes in the genome (Suppl. Figure 6; two-sample two-sided Kolmogorov-Smirnov test for distributions p-value >0.05). Thus, we conclude that there is no significant difference in colocation of predictions of either HMCan or CCAT with the regions of gain or loss and thus the location of predictions are not the cause of the better performance of HMCan as compared to CCAT.

Comparison of HMCan, SICER and MACS.

The MACS and SICER tools demonstrated a bias towards high copy number regions (Figures 3 and 4). Thus, in the main manuscript we excluded these tools from the comparison with HMCan. Here, we would like to report how these tools behave on the experimental H3K27me3 cancer dataset in comparison to HMCan.

To detect the H3K27me3 mark, we ran SICER and MACS with the parameters learned from the simulated data study (these appeared to be the default parameters for MACS and Gap=600 for SICER). We considered all regions detected by SICER or MACS regardless of the score.

We studied the correlation between gene expression and H3K27me3 predictions by HMCan, SICER and MACS in promoter regions. We observed that the expression level of “HMCan-specific” genes was significantly lower than the expression level of “SICER-specific” and “MACS-specific” genes (Suppl. Figure 4; one-tailed Mann-Whitney test p-value < 10^{-16} and p-value = 0.01 for SICER *vs* HMCan and MACS *vs* HMCan, respectively). Since H3K27me3 is a repressive chromatin mark, we conclude that HMCan produced more relevant predictions on this experimental dataset than MACS and SICER.

We also calculated the H3K27me3 signal distribution around TSSs of coding genes (Suppl. figure 5). The resulting distribution was more symmetric around TSSs for SICER and for MACS than for HMCan. The characteristic valley at the TSS was distinctly present in the distribution of H3K27me3 sites predicted by the three tools.

We checked whether MACS and SICER were able to identify H3K27me3 sites previously validated by qPCR in the CL1207 cell line (Vallot *et al.*, 2011). We observed that SICER and MACS were much less sensitive in peak detection in these regions than CCAT and HMCan (Suppl. Table 3). Although both SICER and MACS detected some signal in the *PLCD1-DLEC1* and *HOXD* loci, they failed to detect peaks overlapping most of the sites tested by the qPCR. The low prediction sensitivity of SICER and MACS in these regions may be due to the copy number status of the loci (*PLCD1-DLEC1*: normal*; HOXD*: loss) and the fact that both SICER and MACS are more sensitive in the regions of high copy number.

SUPPLEMENTARY FIGURES

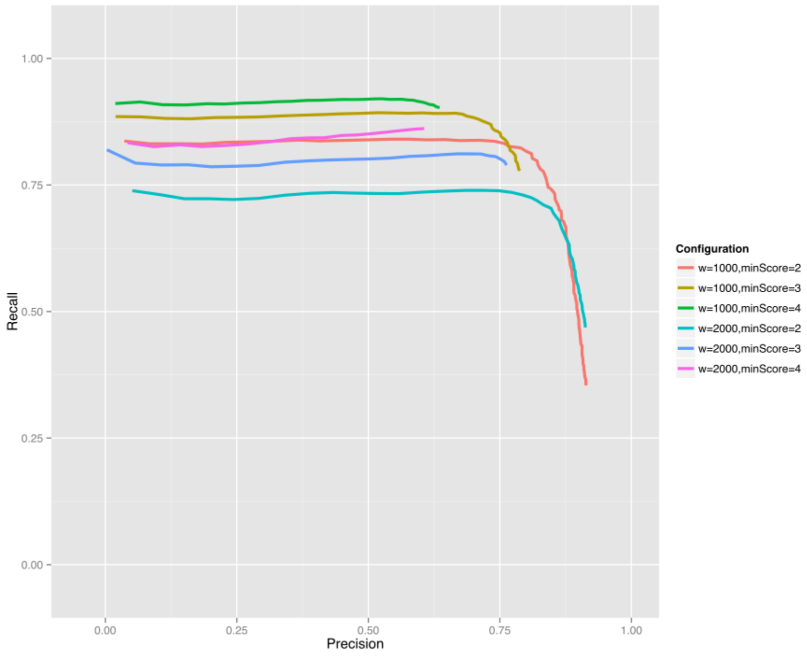

Supplementary Figure 1. Accuracy of CCAT predictions with different parameters: *minScore* and *Window* (w). The "best" combination of parameters corresponds to minScore = 2, w = 1000 (red).

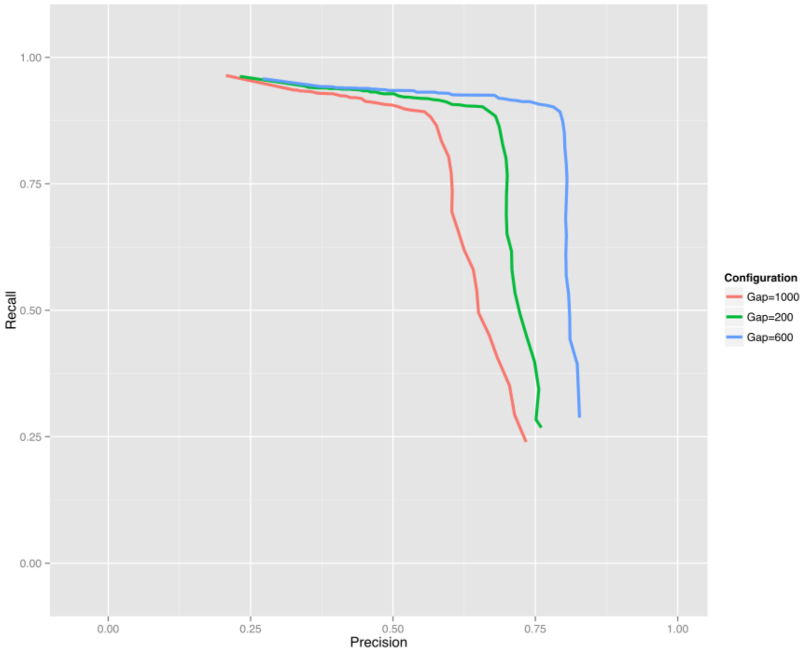

Supplementary Figure 2. Accuracy of SICER predictions with different values of the *Gap* parameter. Best performance is achieved with Gap=600 (blue curve).

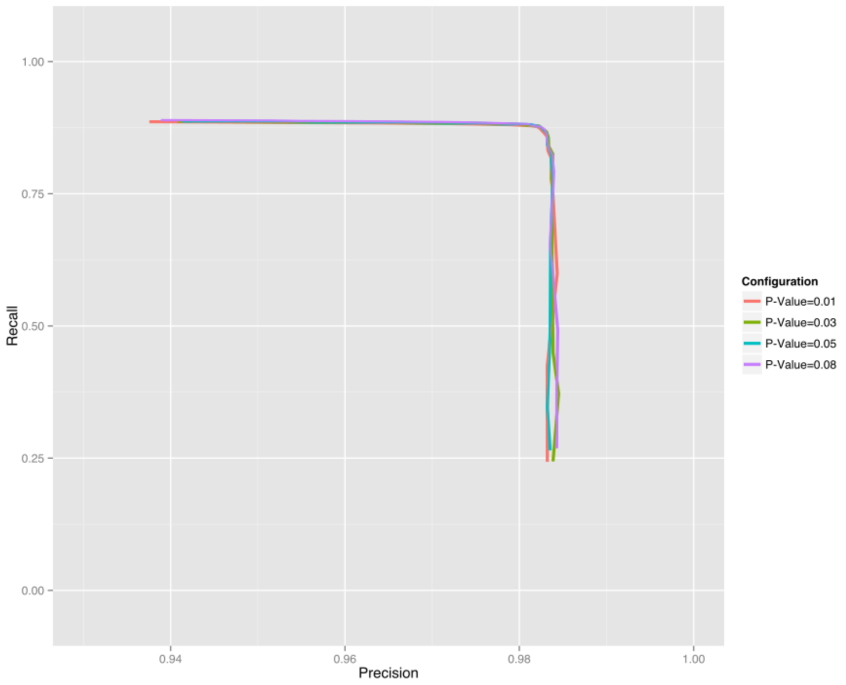

Supplementary Figure 3. The iterative HMM shows stable performance on simulated data regardless the initial value of the threshold on exact Poisson test p-value used for definition of initial transition and emission probability distributions. "Precision *vs* Recall" curves correspond to final HMCan predictions obtained using different initial parameters: p-value thresholds (red = 0.01; green = 0.03; turquois = 0.05; purple = 0.08).

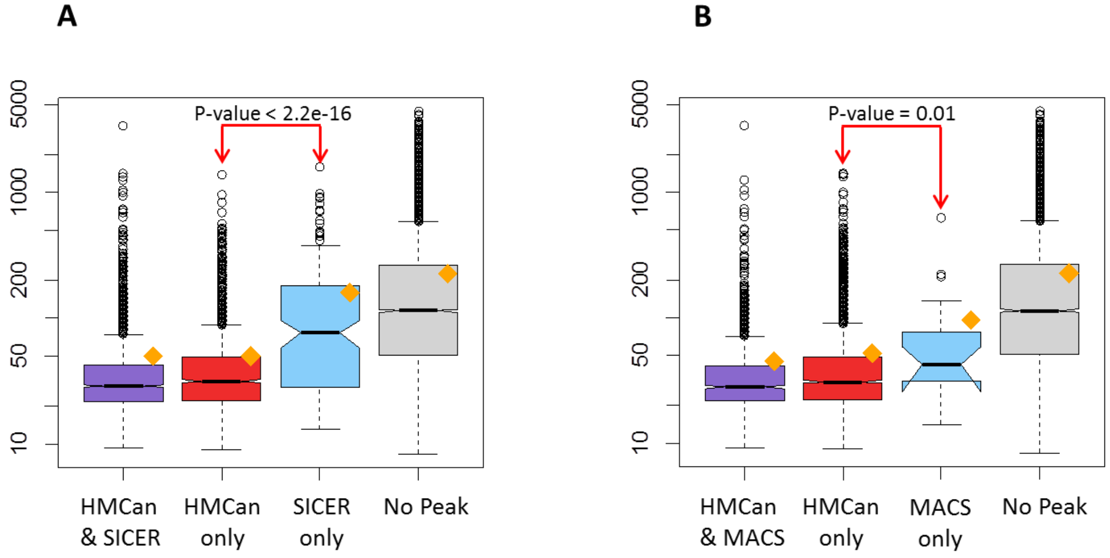

Supplementary Figure 4. Genes with an H3K27me3 mark predicted by HMCan in the promoter regions (TSS±1kb) tend to have lower expression than genes with this mark predicted by SICER (**A**) or MACS (**B**). The graph shows the distribution of gene expression values after RMA normalization for the following gene categories: genes with the promoter H3K27me3 mark predicted by (1) both HMCan and (SICER, MACS), 2478 and 1602 genes, (2) HMCan only, 2003 and 2879 genes, (3) by (SICER, MACS) only, 149 and 19 genes, and (4) by none of the tools, 13068 and 13198 genes. The orange dot shows the mean expression value. The black line shows the median and the boxes are plotted between the 1st and the 3rd quartiles. P-value is shown for one-tailed Mann-Whitney test.

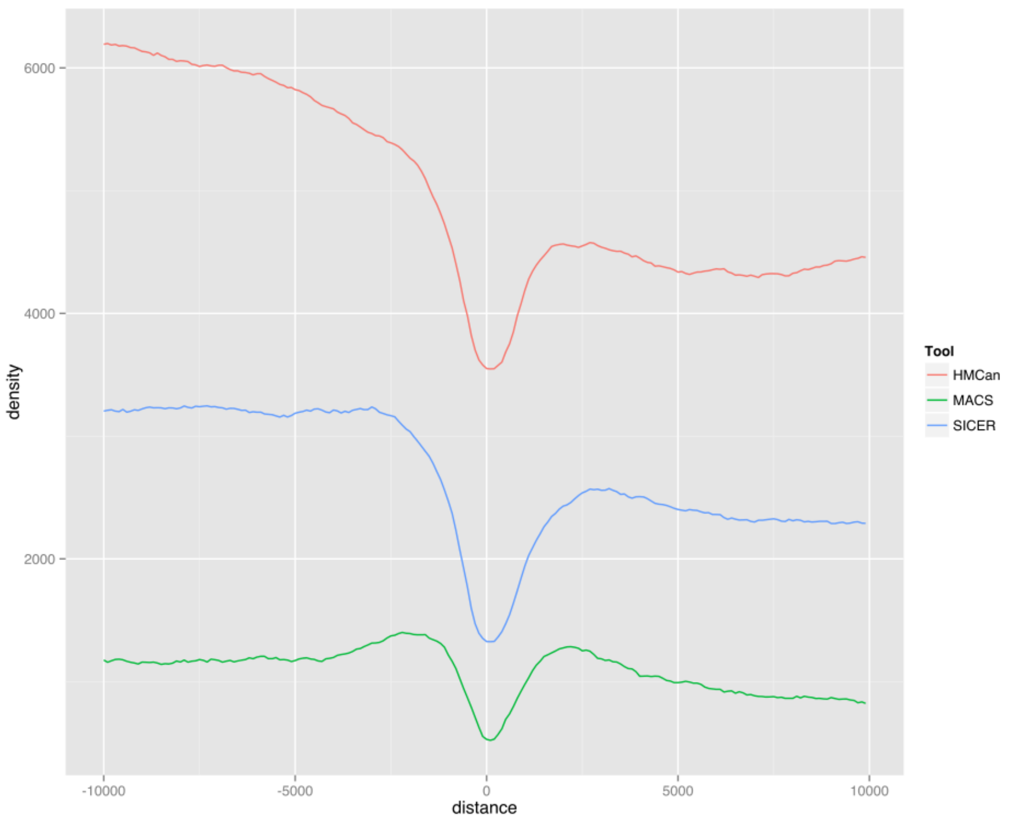

Supplementary Figure 5. Density of peaks detected by HMCan, MACS and SICER around all gene TSSs for the H3k27me3 histone mark in the CL1207 cell line.

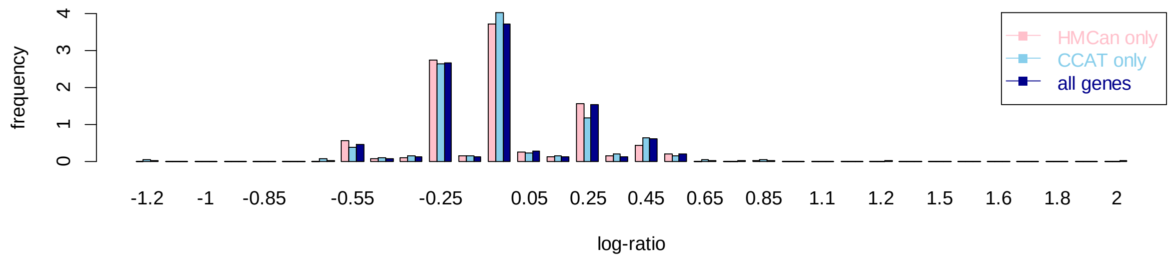

Supplementary Figure 6. Log-ratio values of genomic regions containing genes with H3K27me3 sites in the promoter region predicted by HMCan only (pink), by CCAT only (light blue) and log-ratios of genomic regions containing all RefSeq genes in the CL1207 human bladder transitional cell carcinoma cell line (dark blue). Log-ratios are estimated by GAP (Popova *et al.*, 2009). The "zero"-level corresponds to normal copy number status; values lower than -0.1 correspond to genomic losses; values higher than 0.1 correspond to genomic gains (see Suppl. Figure 13).The three distributions are not significantly different from each other (Two-sample two-sided Kolmogorov-Smirnov test p-values >0.05).

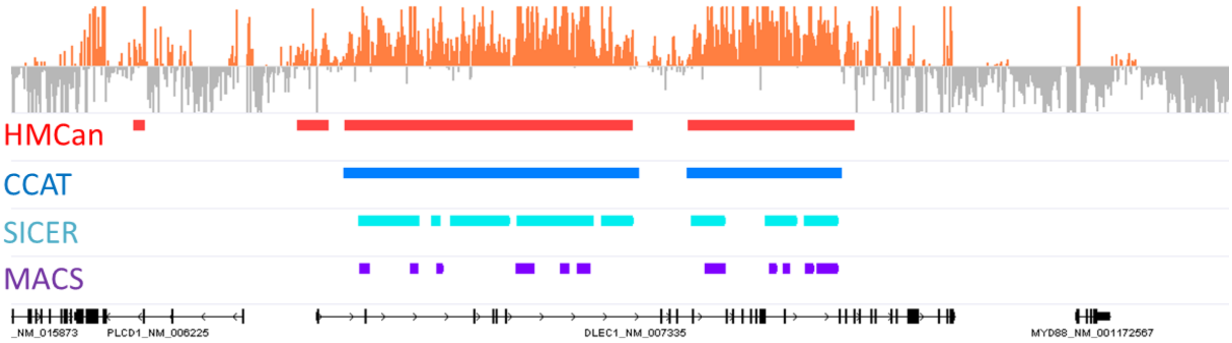

Supplementary Figure 7. HMCan is the only tool among tools tested that was able to detect H3K27me3 signal in the proximity of TSS of the *DLEC1* gene. This region was previously validated by qPCR in the studied cell line. The graph shows H3K27me3 density profile normalized by HMCan and the regions predicted as bearing H3K27me3 by HMCan, CCAT, SICER and MACS around the *DLEC1* gene.

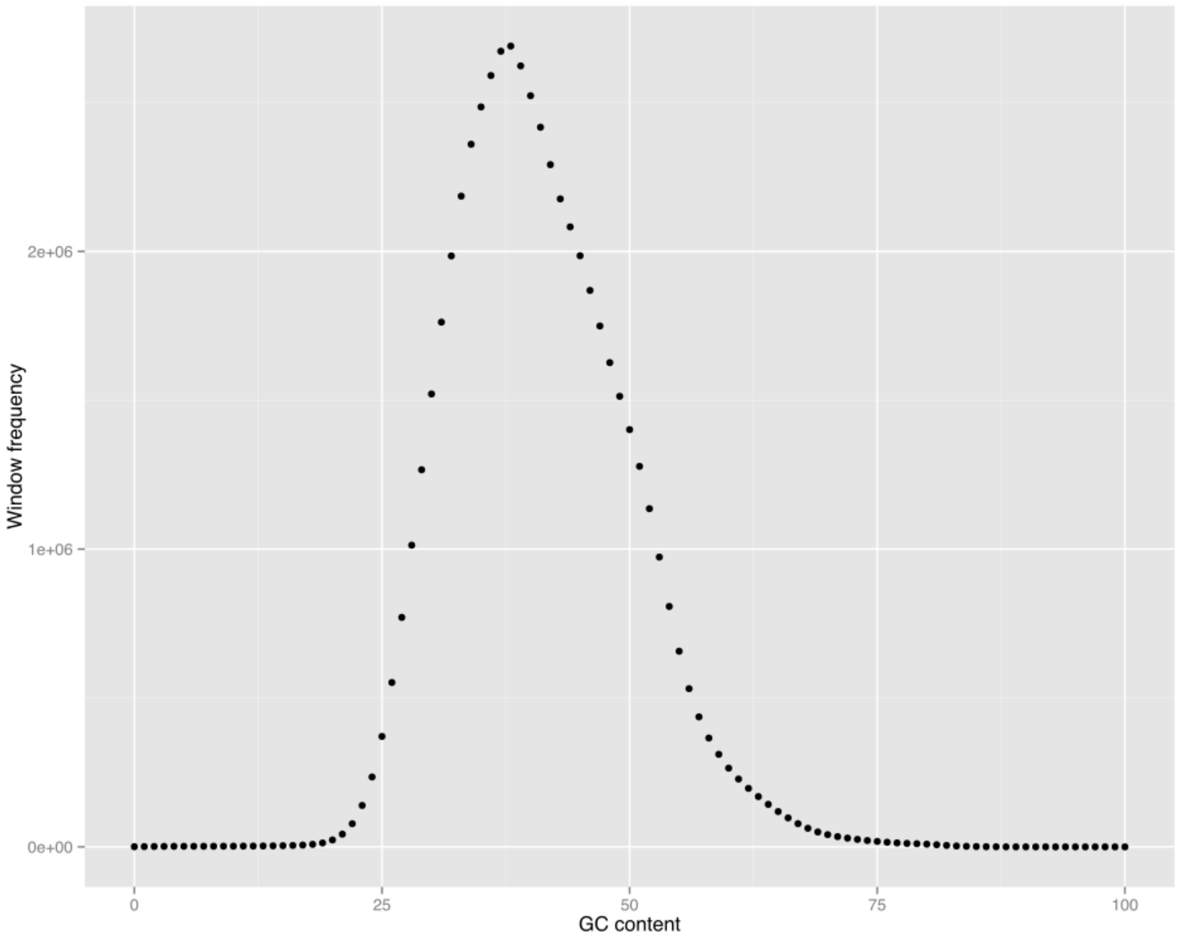

Supplementary Figure 8. Distribution of GC-content values in 300-bp windows in the human genome (version hg19). In the frequency calculation, we used a sliding window of 300bp with an overlap of 50bp. We did not consider windows with undefined nucleotides ("N").

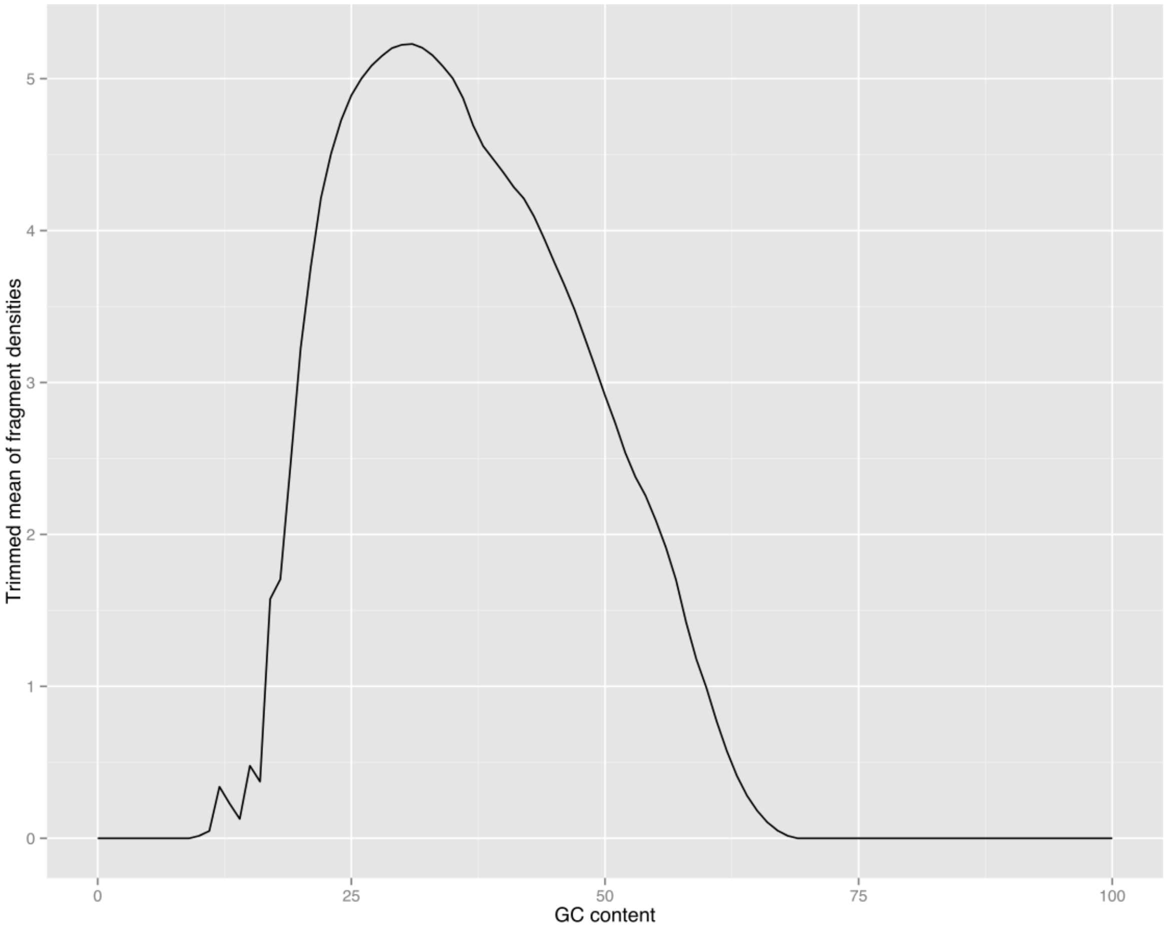

Supplementary Figure 9. Distribution of 10%-trimmed means of fragment densities for each value of GC-content in the control (input) dataset for the human bladder cancer cell line CL1207. Departure from the bell-shaped distribution is visible for GC-content < 0.23.

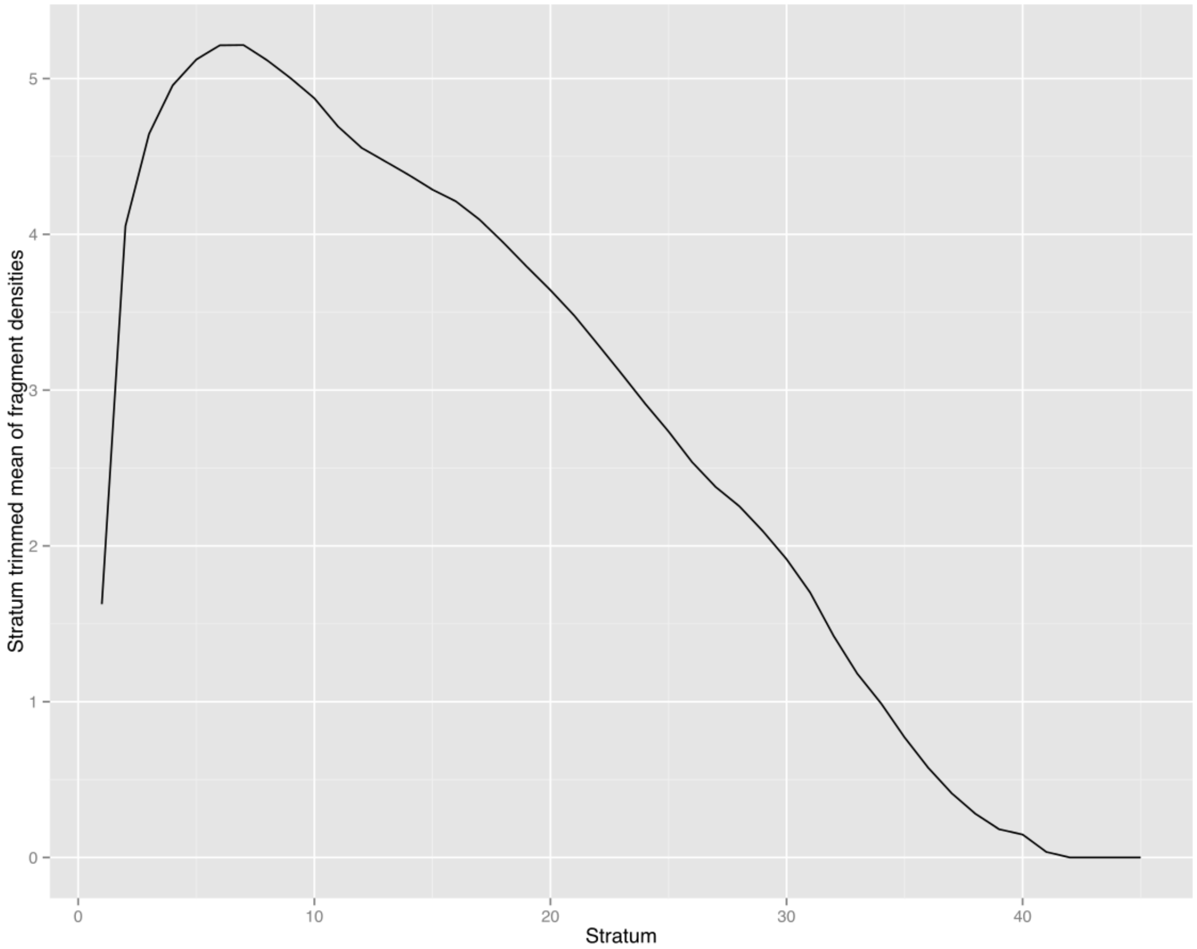

Supplementary Figure 10. Distribution of 10%-trimmed means of fragment densities for each value of GC-content in the control (input) dataset for the CL1207 when performing un-uniform binning. There is no visible fluctuation of the density values from the bell-shaped distribution. GC-content strata: [0,20), [20,22), [22,24),…, [34,36), [36,37), [37,38), …, [64,65), [65,66), [66,68),…, [72,74), [74,100].

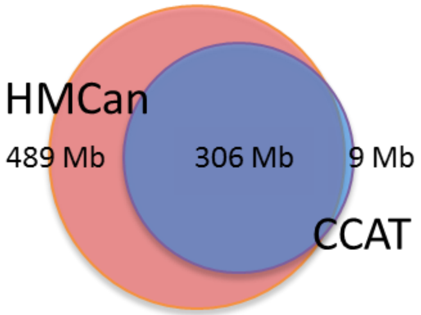

Supplementary Figure 11. Venn diagram showing base pair overlap in the HMCan and CCAT predictions with thresholds 10 and 3.35, respectively. Numbers show the total number of nucleotides in predicted regions, Jaccard similarity coefficient 0.38.

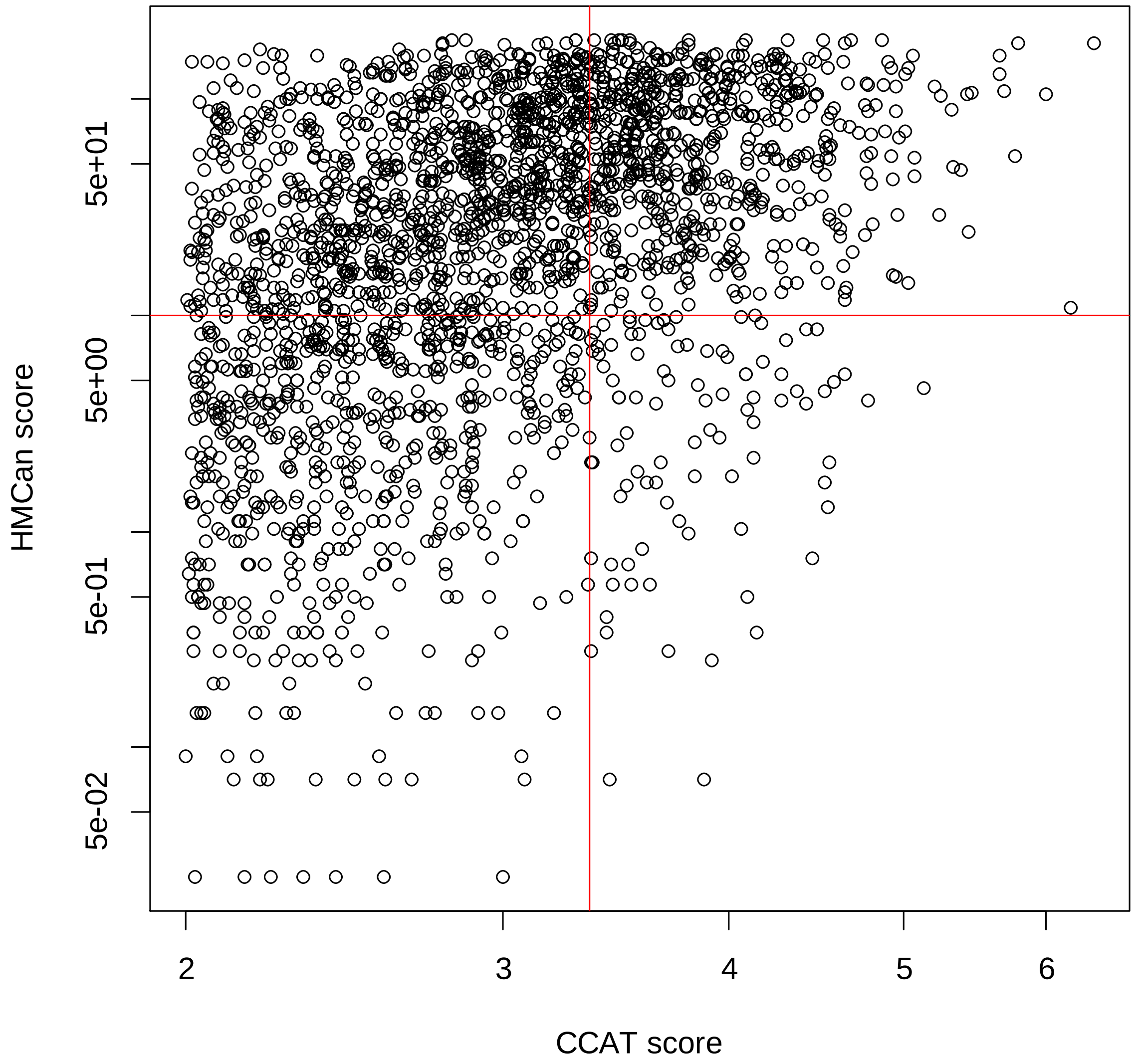

Supplementary Figure 12. Scatter plot (in log-scale) for respective scores of peaks predicted by HMCan and CCAT in the vicinity of gene TSS (±1 Kb) for the H3K27me3 dataset. Only TSSs with H3K27me3 predicted by both tools are considered. Spearman's rank correlation rho 0.43. Red lines correspond to “best” threshold values according to simulations.

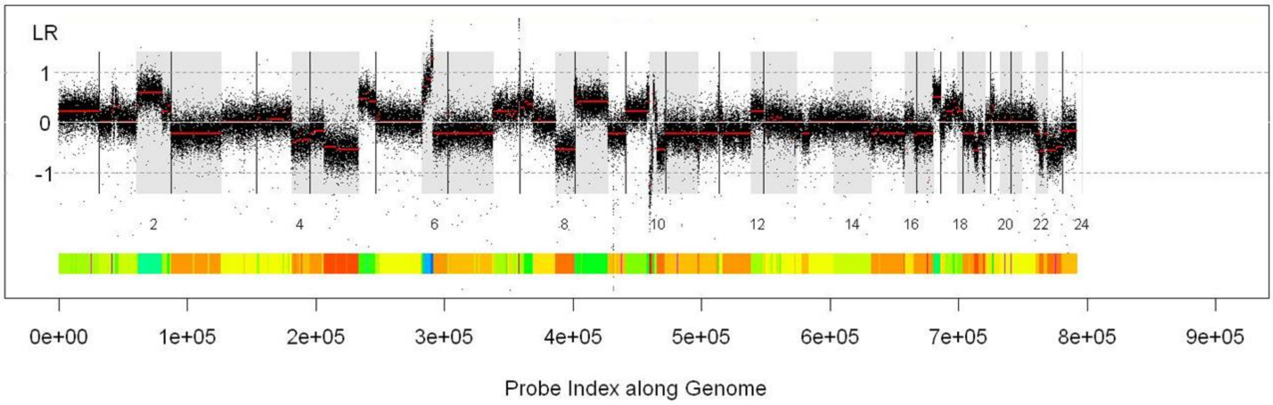

Supplementary Figure 13. Log-ratio profile calculated by GAP (Popova *et al.*, 2009) for the CL1207 human bladder transitional cell carcinoma cell line using SNP array technology.

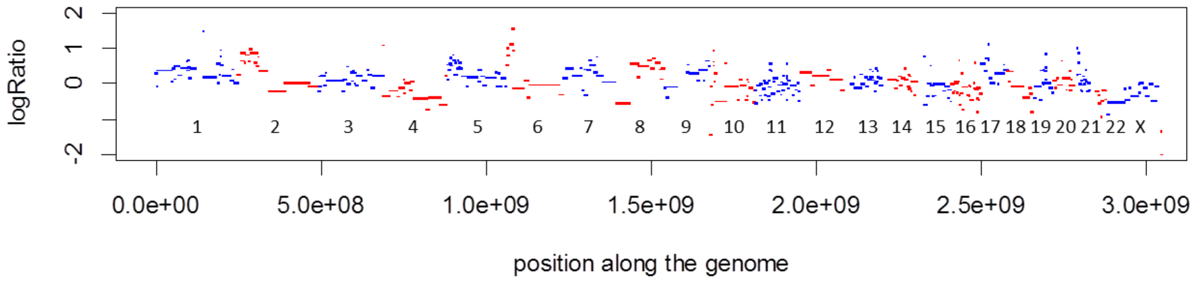

Supplementary Figure 14. Log-ratio profile calculated by the FREEC module within HMCan using the control (input) sample for the CL1207 human bladder transitional cell carcinoma cell line.

SUPPLEMENTARY TABLES

Supplementary Table 1. Repartition of a simulated histone modification signal on human chromosome 1. To test prediction accuracy of our method, we generated strong and weak signals covering all or several alleles, respectively.

Copy number	Number of covered alleles	Start	End
0	0	0 Mb	40 Mb
1	1	40 Mb	90 Mb
2	1	90 Mb	125 Mb
2	2	125 Mb	160 Mb
3	1	160 Mb	173 Mb
3	2	173 Mb	186 Mb
3	3	186 Mb	200 Mb
4	1	200 Mb	212 Mb
4	2	212 Mb	224 Mb
4	3	224 Mb	236 Mb
4	4	236 Mb	249 Mb

Supplementary Table 2. Jaccard similarity index between sets of HMCan predictions corresponding to different starting points for iterative HMMs (different thresholds on the exact Poisson test p-values for initial state calling).

P-value	0.01	0.03	0.05	0.08
0.01	1	0.985	0.981	0.976
0.03	0.985	1	0.993	0.985
0.05	0.981	0.981	1	0.988
0.08	0.976	0.985	0.988	1

Supplementary Table 3. Location of predicted peaks for the H3K27me3 mark at the experimentally validated loci (Vallot *et al*., 2011); positively validated loci are shown in orange.

Genes	Forward primer 5'->3'	Reverse primer 5'->3'	% H3K27me3 (ChIP-QPCR)	HMCan score	CCAT score	MACS score	SICER score
CTDSPL-VILL	atacatccctcaagtccacc	gctacagaggaaaggcaagt	0.04	0	0	0	0
VILL promoter	ggcacgaccaaataggagagtt	ggggcttacgggaactttttg	0.12	0	0	0	0
VILL	tctaagcaccttctctttggg	tcccagagccagatgaggaa	0.32	0	0	0	0
VILL-PLCD1	gttggtctctccagataccc	cacacacacacgcttcacag	0.1	0	0	0	0
PLCD1	ccaatacaaggtcctcctctc	cactgggagtcatccatacgt	0.38	0	0	0	0
PLCD1 promoter	cgttcgcattcccttctg	ggagtgttgggggtgtgg	0.22	0	0	0	0
PLCD1-DLEC1	ggcaacaaaagcaaaactct	tgagtgctggaagtcaccac	0.18	0	0	0	0
DLEC1 promoter	caatgtgatgttatgctgctg	tttcttacctcccattatccac	0.22	**0.25**	0	0	0
DLEC1	gtggtgagtgtggaggaaaag	cgtcccagtccttatgctaaa	0.38	**187.49**	**2.63**	0	0
DLEC1-ACAA1	accacagccactaagataaat	gacccaggctgcttttatct	0.02	0	0	0	0
ACAA1	ccactgccttctcaactttct	caggaaatggaagtcaaggg	0.02	0	0	0	0
ACAA1 promoter	caggaccgcttactgcttg	aatacttccgagacgccc	0.02	0	0	0	0
HOXD9	ctataatcaaatacacctcataaa	gctgtgagcaagcgtgtgtg	0.5	**80.48**	**3.84**	0	0
HOXD9-HOXD8	gatttctgtaatttcttggttg	ttttgttaaatctagcctgtcc	0.18	**12.34**	**3.79**	**58.05**	**164**
HOXD8 promoter	tgggcatgatttccattaaata	ctttgataacgccaggatct	0.5	**12.34**	**3.79**	0	**164**
HOXD8	tatttcaccccgtagacccc	tgttttaatcagcagctcctgg	0.35	**53.67**	**5.07**	**258.12**	**387**
HOXD8-HOXD4	gtcccaggaaggtggatactt	ccaaaatccctacgaaacagg	0.35	**53.67**	**4.19**	0	**257**
HOXD4 promoter	ctcagaggcgagttcaccc	gaaggttggcaaaatcagtgg	0.35	**53.67**	**4.19**	0	0
HOXD4	ctacagtcagtccgacccca	aacctttctgtccacatccc	0.28	**53.67**	**3.77**	0	0
HOXD4-HOXD3	aacctcaaaagcaaggagcat	cgcttggcttcatcagaact	0.32	**53.67**	**4.53**	0	0
HOXD3 promoter	ccaggagccaactgagtctg	aagtgtttgtaagcaagaaccg	0.32	**53.67**	**4.53**	0	0
HOXD3	aggattcagcagacattggag	ctacggacacttacggctaca	0.3	**53.67**	**4.53**	0	0
HOXD3-HOXD1	caaggttgggaacaaatggat	agtagtagaggcaggtgaag	0.37	**53.67**	**4.65**	**59.21**	**191**
HOXD1 promoter	cacgctcccatttaaccttttc	gagagcaggcgttctgccct	0.4	**53.67**	**4.65**	0	0
HOXD1	tccactgctcacccacattct	caagcaactgacagaactgga	0.32	**194.76**	**4.65**	0	**647**
HOXD1-MTX2	cattgctgtgggcagaaaaaa	cttagagagccctttgttgct	0.25	**194.76**	**4.65**	**120.87**	**501**
MTX2	cgtttacaaatgttactcggtt	gaattggctggacggagcta	0.07	0	0	0	0

Supplementary Table 4. Running time of HMCan, SICER, MACS and CCAT on simulated and experimental datasets (using one core of the 16-core Intel Xeon @ 2.67GHz).

Data set	Size of ChIP file	Size of control file	HMCan Running time in minutes	SICER running time in minutes	MACS Running time in minutes	CCAT Running time in minutes
Simulated data	236 MB	234 MB	2	7	4	0.5
CL1207-H3k27me3	3.9 GB	3.9 GB	67	128	32	6
